# Supplementary material for: Patterns of Intron Gain and Loss in Fungi
Source: PLoS Biol. 2004 Nov 30;2(12):e422. doi: 10.1371/journal.pbio.0020422 (PMC532390; doi:10.1371/journal.pbio.0020422)
Supplement: Table S1 — Also available at http://genes.mit.edu/NielsenEtAl/. (4.3 MB ZIP). [file pbio.0020422.st001.zip › NielsenEtAl/html/1182.html]

AN9097.1.NCU07182.1.MG10680.1.FG04476.1


```
 CLUSTAL W (1.82) Multiple Sequence Alignments - Introns Inserted


Sequence 1: NCU07182.1	136 aa
Sequence 2: FG04476.1	136 aa
Sequence 3: MG10680.1	135 aa
Sequence 4: AN9097.1	133 aa
Alignment Length: 136 aa
Number Identitical Residues: 96 aa
Alignment Score (without introns) 4083


MG10680.1 	MAD-DTPVTLRTRKFIRNPLLGRKQMVV2DILHPTRPNISKDELREKLSAMYKATKDQVS
NCU07182.1	MADSDSPVTLRTRKFIRNPLLGRKQMVV2DILHPNRPNISKDELREKLATLYKATKDQVN
FG04476.1 	MADNDSPVTLRTRKFIRNPLLGRKQMVV2DILHPNRANISKEELREKLGSLYKAQKDQIS
AN9097.1  	MAD--APVTLRTRKFIRNPLLARKQMVV2DVLHPNRANVSKDELREKLADLYKSNKDQVS
          	***  :***************.****** *:***.*.*:**:******. :**: ***:.

MG10680.1 	VFGLRTQFGGGKTTGFALIYDSPEALQKFEPRYRLVRIGKATKVEKASRQQR1KQRKNRQ
NCU07182.1	VFGLRTQFGGGKTTGFALVYDSPEAMKKFEPHYRLVRVGLASKIEKASRQQR1KQRKNRQ
FG04476.1 	VFGLRTQFGGGKTTGFALVYDSPEAMKKFEPQYRLVRVGLATKAERASRQQR1KQRKNRQ
AN9097.1  	VFGFRTQYGGGKSTGFALIYDSTEALKKFEPRYRLIRIGAAEKIEKPSRQQR1KQRKNRS
          	***:***:****:*****:***.**::****:***:*:* * * *:.***** ******.

MG10680.1 	KTLRGTAKVKGAKAKKDK
NCU07182.1	KTLRGTAKVKGAKPKKEK
FG04476.1 	KTLRGTAKVKGAKAKKEK
AN9097.1  	KKFRGVAKVKGPKKSKD-
          	*.:**.*****.* .*:
```
